# Understanding advance care planning in care homes throughout the COVID-19 pandemic: A critical realist review and synthesis

**DOI:** 10.1177/02692163221137103

**Published:** 2022-11-12

**Authors:** Adam Spacey, Sam Porter

**Affiliations:** 1 School of Health and Society, University of Salford, Salford, UK; 2 Department of Social Sciences and Social Work, Bournemouth University, Bournemouth, UK

**Keywords:** Palliative care, terminal care, residential facilities, pandemics, coronavirus infections, SARS-CoV-2

## Abstract

**Background:**

The COVID-19 pandemic has disrupted advance care planning discussions in care homes, particularly discussions involving relatives and surrogate decision makers. There is a need to collate and examine current evidence to assess the extent of the problem.

**Aim:**

To examine the processes and experiences involved in advance care planning in care homes throughout the COVID-19 pandemic.

**Design:**

A critical realist review and synthesis.

**Data Sources:**

MEDLINE, psycINFO, SCOPUS and CINAHL were searched between December 2019 and May 2022.

**Results:**

Eleven studies were included. Communication difficulties associated with remote technologies meant that care home staff’s concerns about engaging effectively with relatives further exacerbated the emotional toll of dealing with high death rates in circumstances where staff shortages stretched the capacity of those remaining to provide timely advance care planning discussions. The threat of the pandemic tended to encourage earlier and more frequent advance care planning discussions, though this tendency was partially countervailed by the difficulties that some residents and relatives had in engaging with remote communication modes. There was evidence that education and training in advance care planning increased staff’s confidence and readiness to engage in care planning during pandemic conditions.

**Conclusion:**

Results highlight part of the new context facing staff, relatives and residents in care homes, thus providing valuable insight for future intervention development required to maintain and improve the effectiveness of advance care planning in care homes during and beyond the pandemic.

**What is already known about the topic?**

- The COVID-19 pandemic has disrupted advance care planning discussions in care homes.

**What this paper adds?**

- The introduction of remote communication in circumstances where death was perceived to be close was a tendency for relatives to have more frequent care planning conservations.
- There was an increase in the number of residents and relatives deciding against the option of hospitalisation, hospitals being associated with a higher probability of infection and a lonely death.
- Education and training were found to improve care home staff’s confidence and preparedness for advance care planning during pandemic conditions.

**Implications for practice, theory or policy**

- Results indicate that targeting education and training on managing and developing holistic discussions remotely, the symptoms and trajectories of decline associated with COVID-19 and supporting relatives’ emotions and expectations in cases of restricted care home visits is needed to improve and maintain effective care planning during and beyond the pandemic.

## Introduction

Residents living in care homes often have multiple complex conditions which increase their vulnerability to serious complications and mortality from COVID-19.^1^ Analysis of the national datasets of 25 counties shows that mortality in care homes was on average 30% of total COVID-19 deaths.^2^ Despite the rollout of vaccination programmes,^3^ COVID-19 infection rates have remained high in many developed countries due to evolving and highly transmissible variants and the lifting of restrictions such as social distancing.^4,5^ Thus, given this risk to residents, it is important for care home staff to be aware of residents’ care preferences in the event of COVID-19 infection. Advance care planning is one process care home staff can use to help align care to residents’ preferences.^6^

An international panel consisting of members from Europe, North America and Australia collectively describe advance care planning as a process of ‘enabling individuals to define goals and preferences for future medical treatment and care, to discuss these goals and preferences with relatives and healthcare providers, and to record and review these preferences if appropriate’.^7^ Topics of discussion can range from treatment preferences, prognosis and bereavement support.^8,9^ Furthermore, given the high prevalence of residents living in care homes with some level of progressive cognitive impariment,^10,11^ the care planning process can include choosing a trusted person or persons who can make decisions about medical care in the event capacity to make such decisions is lost.^12^ Sustaining these ongoing discussions which allow residents and relatives to share care preferences with care home staff during the pandemic and beyond is important, given these discussions have been found to increase the quality of care provided and proportion of residents dying in their preferred place of death.^13^

It has been well acknowledged that challenges in the provision of advance care planning have existed long before the COVID-19 pandemic, such as a lack of engagement and reluctance from care home staff initiating conversations,^9,14^ insufficient knowledge and skills of care home staff,^15,16^ and low uptake of care planning particularly for residents with some level of cognitive impariment.^17^ However, emerging evidence suggests that the pandemic has further disrupted advance care planning with decreased levels of care planning in care homes and fewer residents being able to express their wishes.^18^

Thus, given the arguably increased importance of advance care planning and the ongoing pandemic in care homes, there is a need to synthesise current evidence to examine the processes and experiences involved in advance care planning in care homes throughout the COVID-19 pandemic. Identification, collation and evaluation of effective advance care planning practices as well as barriers faced during the pandemic can be used to support and inform advance care planning practice in care homes during and beyond the pandemic. In this review, the term ‘care home’ refers to both residential and nursing homes, which provide food and board, 24-h care cover and assistances where required with activities of daily living. Nursing homes additionally provide care by registered nurses.

## Methods

### Aims

To examine the processes and experiences involved in advance care planning in care homes throughout the COVID-19 pandemic.

Objectives:

- To identify mechanisms embedded in the social and organisational context.
- To identify mechanisms embedded in advance care planning interventions impacting on behavioural patterns.
- To examine the ways in which different individuals respond to advance care planning during the pandemic.
- To identify outcomes in terms of rates of behaviour and experiential consequences.

### Design

Given the need to evaluate and understand the complexity of factors influencing the application of advance care planning during the pandemic, this review adopted a critical realist design and synthesis, which assumes that the outcomes of interventions result from the interaction of a plurality of causal mechanisms.^19,20^ Mechanisms can be described as natural, social or individual powers that generate tendencies in events. While they may not be observable, their influence can be retroduced from what is observed.^19,21^

While holding many of the basic assumptions of realist evaluation,^22^ critical realist evaluation differs in some important respects. In contrast to the realist evaluation categories of ‘context’ and ‘mechanism’, critical realist evaluation posits three categories:

Contextual mechanisms: these operate in the social contexts into which interventions are introduced.

Intervention mechanisms: these are the social mechanisms embedded in interventions with the aim of replacing what have been identified as undesirable behavioural patterns with more desirable ones.

Human agency: these are the responses of stakeholders (both those implementing interventions and those receiving them) to interventions within specific social contexts.

The change from ‘context’ to ‘contextual mechanisms’ reflects the recognition that the contexts in which interventions are introduced actively influence the outcomes produced and that the mechanisms by which they exert influence need to be specifically identified. The change from ‘mechanisms’ to ‘intervention mechanisms’, which assumes that social mechanisms are embedded in both context and intervention, is a corollary to the first revision. The addition of ‘agency’ is based on the critical realist assertion that the powers of individuals to engage in meaningful action are categorically different from the mechanisms embedded in social structures. The reasons that people have for acting in the ways that they do are not the same things as the external influences that are brought to bear on their reasoning,^22^ though they have a reciprocal relationship. While structures supply agents with directional guidance and shape the patterns of social action, they are in turn either maintained or transformed by human agency.^23^ Thus, their relationship is one of temporal iteration. Distinguishing between social and agential mechanisms once again facilitates a clearer and more specific analysis than the portmanteau category of ‘mechanism’ allows for.

The development and implementation of interventions can be viewed in this light. They involve agents perceiving a problem in the current social configuration, hypothesising about the changes required rectify it, and then introducing programmes containing novel countervailing mechanisms with the aim of effecting those changes. However, because of the multiplicity of other mechanisms embedded in the social context and agents’ volition, what transpires will not necessarily be what was envisaged. This new social configuration then faces agents as ‘an objective influence’^23^ (p. 196), which will again be subject to agents’ activities that will result in its reproduction or transformation.

Critical realist evaluation’s approach to outcomes is also distinctive. On the grounds that ‘evaluative descriptions’ of social facts involve both reason and values,^24^ in addition to identifying changes in rates of behaviour over time (which is the focus of realist evaluation), it also seeks evaluative evidence in terms of interventions’ effects on the flourishing or suffering of those exposed to them.^19^

Although realist reviews do not traditionally aim to exhaustively search the literature,^25^ a systematically constructed search was deemed necessary to capture as many relevant mechanisms as possible within currently published literature. This review was reported in accordance with the Realist and Meta-narrative Evidence Synthesis: Evolving Standards (RAMESES).^20^

### Search strategy

The authors’ previous research in the field of advance care planning^18,26^ as well as preliminary searches were used to develop the search terms. Four databases were searched: SCOPUS, MEDLINE, CINAHL and psycINFO for English language papers published between December 2019 and 28th May 2022. This date range was selected as this review aims to elicit data describing and evaluating advance care planning in care homes from the start of the COVID-19 outbreak to present day pandemic conditions. Moreover, as the impact of pandemic conditions on advance care planning have been experienced in care homes internationally, no location restrictions were placed on the search.

**Figure 1.**
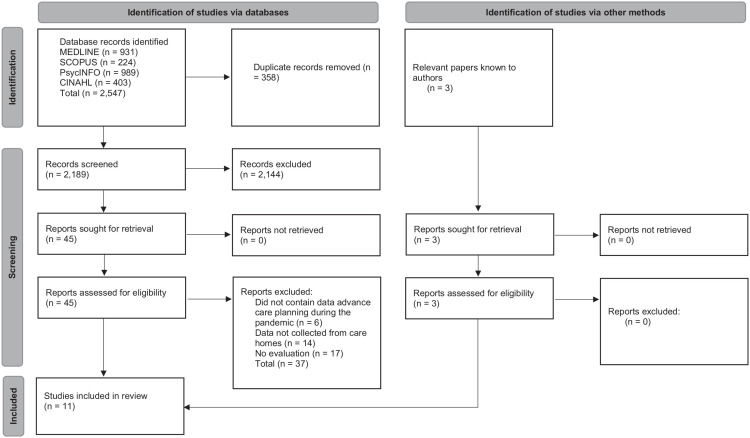
Document flow diagram.

The strategy of search as well as Boolean teams used are detailed in Table 1. The search also used forward and backward citation searching of relevant policy documents, studies and grey literature. Papers already known to the authors were also included and detailed in Figure 1.

**Table 1.** Search strategy.

Element	Alternatives	
1. ‘Advance care plan*’	‘Care plan*’ Dying Death* ‘End of life care*’ ‘Anticipatory care plan*’ “End of life discussion” ‘Advance directive*’ ‘Do not resuscitate’ DNR	‘End of life plan*’ ‘Serious illness plan*’ ‘Shared decision marking’ ‘Surrogate decision maker’
2. ‘Care home*’	‘Nursing home*’ ‘Nursing care home*’ ‘Residential home*’ ‘Residential care home*’ ‘Long-term care facili’	‘Rest home*’ ‘Respite care’ ‘Long-term care’ ‘Resident*’ ‘Respite care’
3. ‘COVID-19’	Coronavirus Pandemic SARS-CoV-2 Lockdown* Quarantine Social distanc*	
Boolean Operators	1. ‘Advance care plan*’ OR ‘Care plan*’ OR Dying OR Death* OR ‘End of life care’ OR ‘Anticipatory care plan*’ OR ‘End of life discussion’ OR ‘Advance directive*’ OR ‘Do not resuscitate’ OR DNR OR ‘End of life plan*’ OR ‘Serious illness plan*’ OR ‘Shared decision marking’ OR ‘Surrogate decision maker*’	
	2. ‘Nursing home*’ OR ‘Nursing care home*’ OR ‘Residential home*’ OR ‘Residential care home*’ OR ‘Long-term care facili’ OR ‘Rest home*’ OR ‘Respite care’ OR ‘Long-term care’ OR ‘Resident*’ OR ‘Respite care’	
	3. COVID-19 OR Coronavirus OR Pandemic OR SARS-CoV-2 OR Lockdown* OR Quarantine OR ‘Social distanc*’	

### Inclusion and exclusion criteria

Title and abstract screenings were conducted by A.S. Relevant articles were then subject to full text screening against the eligibility criteria detailed in Table 2 by A.S and S.P.

**Table 2.** Eligibility criteria.

Inclusion category	Description
Population	Studies must include staff who have been involved in advance care planning with residents and their significant others in care homes during the COVID-19 pandemic.
Intervention	Studies must include data on advance care planning during the COVID-19 pandemic in care homes.
Setting	Studies must include data on advance care planning collected from care homes during the COVID-19 pandemic.
Comparator/outcome	Studies which report advance care planning practices throughout the COVID-19 pandemic in care homes compared to pre-pandemic practice.
Publication	Papers must be peer reviewed published between December 2019and May 2022. Non-peer reviewed papers, book chapters, commentary and opinion pieces and abstracts were excluded.

### Data extraction

A.S. carried out data extraction which involved extracting the data from the included studies into a Microsoft word document. The main features of each article were extracted which included the title, country of data collection, methodology, causal data and results/outcomes. Data extraction was cross-checked by S.P.

### Quality assessment

Independent quality assessment was carried out the two authors A.S and S.P using the Mixed Method Appraisal Tool (MMAT).^27^ The quality of the article selection was screened against the criteria set out in the MMAT. Any disagreements were discussed between the two authors till an agreement was reached. Each of the studies were graded from 0% to 100% with 0%‒20% being (very low), 20%‒50% (low), 50%‒70% (moderate) and 70%‒100% (high). No studies were excluded based on quality, and all the included studies were graded high quality. See Supplemental File 1.

### Data synthesis

We conducted a critical realist synthesis of the included studies.^19,20^ A.S and S.P. coded verbatim sections of the extracted against the four critical realist evaluation categories: contextual mechanisms, intervention mechanisms, human agency and outcomes. Specifically, data were coded and grouped in accordance to how advance care planning worked during the pandemic (intervention mechanisms), the influence of context (contextual mechanisms), and how those involved responded, experienced, and behaved (human agency). The coded data were then collated and identified patterns were arranged into subthemes and themes. Key themes were recorded which identified and described the underlying processes and causal mechanisms of importance for explaining advance care planning outcomes during the pandemic.

## Results

2189 unique records were initially retrieved from database searching, 2144 records were excluded following title and abstract screening. The remaining 45 full-text articles were screened, leaving eight articles which met the inclusion criteria. Relevant papers known to the authors identified an additional three articles. Eleven articles were included in total. Figure 1 details this searching processes.^28^

### Overview of included studies

Of the included papers, six were qualitative,^29–34^ one used a mixed method design^35^ and four used quantitative methods.^36–39^ The studies collected data from seven countries which included the UK (*N* = 3),^31,34,39^ Australia (*N* = 1),^35^ Netherlands (*N* = 1),^33^ Italy (*N* = 1),^30^ Sweden (*N* = 1),^36^ Canada (*N* = 2)^29,32^ and the United States of America (*N* = 2).^37,38^ Participants consisted of care home staff, managers, physicians, residents, surrogate decision makers and nurse practitioners. Qualitative methods included semi-structured qualitative interviews, and online questionnaires. Quantitative methods included analysis of electronic medical records, palliative care registers and case note analysis.

Although all the included studies contained data on advance care planning during the pandemic, the goals and foci within each study varied. *N = 3* studies focused on clinical decision making^37–39^*N = 4* focused on education and training.^29,32,34^*N = 3* studies explored stakeholders’ experiences of providing advance care planning during pandemic conditions and the use of remote communication.^33,31,35^*N = 1* study focused on the number of advance care planning discussions offered during the pandemic.^36^ See Table 3 for a breakdown of each included study and a summary of the extracted causal data.

**Table 3.** Details of the included studies.

Author(s)	Aim	Study	Method(s)	Country	Critical Realist categories	Results/outcomes	Quality score
Vellani et al.^29^	To explore the role of nurse practitioners in facilitating a dignified death for long term care home residents while also facing increased pressures related to the COVID-19 pandemic.	Qualitative	A purposive sample of 14 nurse practitioners working in long term care homes was recruited. Data were generated using semi-structured interviews and examined using thematic analysis.	Canada	*Intervention mechanisms*: Use of nurse practitioners to support/mentor care home staff engage in difficult care planning conservations. *Contextual mechanisms*: Restrictions preventing relatives from monitoring and seeing residents. Lack of resources conducive to timely care planning. *Agency*: Participants demonstrated a recognition and openness towards care planning during the pandemic.	Three categories were derived: (a) advance care planning and goals of care discussions; (b) pain and symptom management at the end-of-life; and (c) care after death.	High
Gonella et al.^30^	To explore the difficulties experienced by Italian nursing home staff in end-of-life conversations with family caregivers during COVID-19 pandemic to uncover their educational needs.	Qualitative	A qualitative descriptive study based on inductive thematic analysis was performed. Twenty-one health care professionals across six Italian nursing homes were interviewed.	Italy	*Intervention mechanisms*: Care home staff hypothesised that case based practical training would support care planning conversations during the pandemic. *Contextual mechanisms*: High staff turnover, shortages and workload during the pandemic. *Agency*: Some relatives still viewed death and dying as a taboo subject and were reluctant to talk about supportive and palliative approaches.	Four themes described their experiences of end-of-life conversations: (1) communicating with family caregivers over the overall disease trajectory; (2) managing challenging emotions and situations; (3) establishing a partnership between HCPs and family caregivers; (4) addressing health care practitioner’s communication skills needs.	High
Hockley et al.^31^	The paper explores how care home staff improvised to address this situation during the first wave of the pandemic	Qualitative	In-depth café-style interviews with twenty-one staff were conducted with care home staff to explore creative practices that they introduced.	UK	*Intervention mechanisms*: Technology based communication between care home staff, residents and relatives. *Contextual mechanisms*: Setting up the technology to engage in remote communication was found to be time consuming. *Agency*: Car home staff felt that residents living with dementia had difficulty getting used to communication technology.	Findings reveal the enormous effort by care staff to maintain family connections and the rapid acclimatisation involved working with a number of different on-line platforms, the pulling together of staff from across the care home.	High
McGilton et al.^32^	To understand the nurse practitioner’s roles in optimising resident care and supporting long term care staff during the pandemic.	Qualitative	This exploratory qualitative study employed a phenomenological approach. A purposive sample of 14 nurse practitioners working in long term care homes in Ontario, Canada, was recruited. Data were generated using semi structured interviews and examined using thematic analysis.	Canada	*Intervention mechanisms*: Nurse practitioners provided mentorship and support to care home staff. *Contextual mechanisms*: Higher workload and expectations during the pandemic. Care staff spent longer with residents setting up care plans often in place of medical staff due to visiting restrictions. *Agency*: None evident in study.	Four categories relating to the nurse practitioner’s practices and experiences during the pandemic were identified: (a) containing the spread of COVID-19, (b) stepping in where needed, (c) supporting staff and families, and (d) establishing links between fragmented systems of care by acting as a liaison.	High
Brugge et al.^33^	To explore how physicians in Dutch nursing homes practiced advance care planning during the first wave of the COVID-19 pandemic, and to explore whether and how advance care planning changed during the first wave of the pandemic	Qualitative	Qualitative analysis of an online, mainly open-ended questionnaire on advance care planning among physicians working in nursing homes in the Netherlands during the first wave of the COVID-19 pandemic. Setting and Participants: Physicians in Dutch nursing homes.	Netherlands	*Intervention mechanisms*: Advance care planning discussions during the pandemic led to new topics of discussion related intensive care unit admission, and treatment preferences if infected with COVID-19. *Contextual mechanisms*: Social distancing restrictions led to less face-to-face time/contact with surrogates. *Agency*: Care home staff and relatives were more aware of death and dying and tended to want to avoid hospital admission.	Four main themes evolved: reasons for advance care planning discussion, discussing advance care planning, topics discussed in care planning, and decision making in advance care planning. COVID-19 specific changes in advance care planning indicated by respondents included COVID-19 infection as a reason for initiating advance care planning a higher frequency of planning discussions.	High
Cousins et al.^34^	To develop, implement and evaluate a website intervention for care staff and family members to provide training and information about advance care planning during COVID-19.	Qualitative	The research was a primarily qualitative case study design, comprising multiple UK nursing home cases. Data collection included semi-structured interviews with care staff and family members which were coded and analysed thematically.	UK	*Intervention mechanisms*: A website intervention containing audio-visual training to support care home staff and relatives with advance care planning during the COVID-19 pandemic. *Contextual mechanisms*: For care home staff, computer skills and time were barriers to training. For relatives, no access to technology was a main barrier. *Agency*: For care home staff, an increased willingness to talk about advance care planning was evident. For relatives, feelings of being valued and involved in advance care planning were found.	The website content that was well received. The study demonstrated that high quality online training and information for care home staff and relatives on advance care planning is effective and implementable. Relatives felt more involved and valued in care planning, and staff shown an increased willingness to talk about care planning.	High
Hack et al.^35^	To explore end-of-life experiences for residents who die in residential aged care facilities and their next-of-kin/carers during the COVID-19 pandemic, to identify areas of concern and areas for improvement.	Mixed methods	Prospective single-centre mixed methods research was undertaken involving telephone interview with next-of-kin or carers of residents who died within 30 days of being referred to Austin Health Residential Service during the ‘second wave’ of COVID-19 in Melbourne, Australia, in 2020.	Australia	*Intervention mechanisms*: Technology used to facilitate communication during care planning. *Contextual mechanisms*: Social distancing restrictions preventing on site visits to the home. *Agency*: Relatives highlighted changing their minds about the hospitalisation of their next of kin during the pandemic.	Forty-one telephone interviews were analysed. Major themes identified included: COVID-19 pandemic, communication and technology, death and dying experience, bereavement and grief, and social supports and external systems.	High
Strang et al.^36^	To study whether end of life discussions were offered and to what degree patients were alone at time of death when dying from coronavirus disease 2019 (COVID-19), comparing deaths in nursing homes and hospitals.	Quantitative.	Data from end of life questionnaires and the palliative care register were analysed in the study. All expected deaths from COVID-19 in nursing homes and hospitals were compared with, and contrasted to, deaths in a reference population (deaths in 2019).	Sweden	*Intervention mechanisms*: None evident in study. *Contextual mechanisms*: None evident in study. *Agency*: None evident in study.	Fewer end of life discussions with patients were held compared with deaths in 2019. In comparisons between nursing homes and hospital deaths, significantly fewer in nursing homes had a retained ability to express their will during the last week of life.	High
Berning et al.^37^	To address advance care planning during a COVID-19 outbreak and its impact on the incidence of new do not-hospitalise directives among long-term care residents.	Quantitative.	Residents who acquired a new do not-hospitalise directive during the study initiative were determined using the electronic medical record. Subsequent changes in not-hospitalise directives orders, hospitalisations, and deaths were ascertained by retrospective chart review from the date of new not-hospitalise directives through August 5, 2020	United States of America	*Intervention mechanisms*: Use of technology, redeployment of staff and telephone evaluations. *Contextual mechanisms*: None evident in study. *Agency*: None evident in study.	Following advance care planning discussions, 124/315 (39%) of residents acquired a new not-hospitalise directive. Among residents with new DNH directives, 65/124 (52%) were diagnosed with COVID-19 from April 2, 2020 to May 21, 2020	High
Ye et al.^38^	To describe the care preference changes among nursing home residents receiving proactive Advance Care Planning conversations from health care practitioners during the COVID-19 pandemic.	Quantitative	Health care practitioners conducted advance care planning conversations proactively with residents or their surrogate decision makers at 15 nursing homes in a metropolitan area of the southwestern United States between April 1, 2020, and May 30, 2020. Descriptive data analyses identified significant changes in resident care preferences before and after advance care planning conversations.	United states	*Intervention mechanisms*: Pro-active reviewing and ongoing care planning discissions with residents and surrogates during the pandemic. *Contextual mechanisms*: None evident in study. *Agency*: More residents and surrogates preferred to avoid admission to hospital when planning care.	Before the most recent advance care planning discussion, 361 residents were full code status, and the rest were Out of Hospital Do Not Resuscitate. Of the individuals with Out of Hospital Do Not Resuscitate, 188 residents also chose Do not hospitalise. After the advance care planning conversation, 88 residents opted to change from full code status to Out of Hospital Do Not Resuscitate, thereby increasing the percentage of residents with Out of Hospital Do Not Resuscitate from 63% to 72%.	High
Jones et al.^39^	To evaluate the clinical presentation, management, care planning and clinical decision-making, and after death care of care home residents who died due to COVID-19	Quantitative	Clinical records of 136 in care homes were reviewed by a General Practitioner reviewer using a standardised template. These were then reviewed by a multidisciplinary panel to identify themes.	UK	*Intervention mechanisms*: The content of advance care plans and the clinical decision making of care home staff was explored during the pandemic. *Contextual mechanisms*: None evident in study. *Agency*: None evident in study.	90% of residents had a record of Do Not Attempt Cardiopulmonary Resuscitation decision, but only 46% had documented advance care planning.	High

Results are organised according to the critical realist evaluation categories of contextual mechanisms, intervention mechanisms, human agency and outcomes.

### Contextual mechanisms

The COVID-19 pandemic generated novel biological and social contextual factors in care homes. They included the combined effects of a highly transmissible and virulent virus and the spatial confinement in close proximity in relatively closed institutions of groups of people with greater than normal vulnerability to infection.

This new context had direct consequences upon advance care planning, in that the novel symptoms and trajectories of decline associated with COVID-19 challenged the capacity to plan for future care.^29,30,35^ They also had indirect consequences. Firstly, actions taken to respond to the threat they posed, notably the introduction of social distancing, altered the organisational context for advance care planning.^30,33^ Secondly, it was not only residents who were vulnerable to the virus. High levels of staff sickness led to shortages which challenged staff’s capacity to fulfil required functions including advance care planning.^30–32^

#### Social distancing: Workloads and expectations

It was evident that reduced or no visiting allowances for relatives and significant others due to social distancing requirements increased the workloads and expectations of staff involved in advance care planning.^30,32,33^ Specifically, several studies noted that lack of regular visits, which would have allowed relatives to monitor residents and notice any deterioration, meant that they depended much more on care home staff in advance care planning discussions.^30,33^ Hack et al.^35^ found that social distancing particularly affected residents living with dementia due to the lack of physical touch and presence, and non-English speaking residents who relied on visiting relatives to act as interpreters.

Conversely, the ease of remote communication, which enabled relatives to get in touch without having to travel to the home appeared to be facilitative, as relatives could be more easily and frequently be involved in advance care planning.^30,33^

Social distancing restrictions also impacted on external service staff involved in care planning, such as General Practitioners.^32,33^ For example, in one study care home staff described themselves as being the doctors’ eyes and ears as more care home staff had to evaluate residents themselves in the absence of in-person GP visits.^32^ Furthermore, the postponements of multidisciplinary meetings, again seemed to result in care home staff being over-relied on.^33^ However, social distancing and measures to limit the spread of COVID-19, also meant that care home staff had to don PPE for each resident which further added to the time and workload required to have face-to-face anticipatory conversations.^33^ The increased workloads and expectations caused by the changes in practice was a consistent finding, regardless of country.^31–33^

#### Staff sickness and shortages

The increases in workload were further compounded by increased staff sickness and shortages due to the pandemic.^30–32^ Studies reported that staff were having to work far beyond their ‘normal’ working hours and take on additional roles to make up for staff absences, with fewer care home staff available to keep up timely care planning assessments.^29,31^ Staff sickness and absence appeared to hit advance care planning particularly hard during the first wave of the pandemic when residents required more high intensity care, and the trajectories of decline were less well known.^29,30^ However, the data also suggest longer term impacts. Specifically, Cousins et al.^34^ report that the most significant barrier to training was care home staff not having the time to engage, and others having to complete the training outside of their working hours.

### Intervention mechanisms

This section identifies and describes the generative mechanisms hypothesised to promote behaviour conducive to supporting advance care planning throughout pandemic conditions.

Two primary types of interventions designed to respond to the novel contexts that resulted from pandemic conditions were identified in the literature. The first involved the development of new modes of remote communication, especially between staff and relatives and surrogates, to replace face-to-face interaction. While this digital technology contained mechanisms that facilitated the convenience and frequency of interaction, it lacked the non-verbal communicative mechanisms embedded in face-to-face interactions. The second involved the training and education of staff in skills related to the technology of remote communication and knowledge of COVID-19 and its care. The mechanisms of skill development and knowledge exchange embedded in these programmes generated a tendency for staff to be more competent and confident.

#### Modes of delivery

It was apparent that the introduction social distancing in response to the pandemic changed how care planning discussions were conducted, with a marked increase in the use of digital technology to facilitate communication. Although face-to-face discussions still occurred between care home staff and residents; relatives and surrogates were often involved in these discussions remotely using video or phone calls due to visiting restrictions.^29,31,33,35,36,38^ Several benefits resulted from the adoption of remote communication compared to face-to-face discussions, which included easier and more frequent access to relatives, being able to schedule calls and being able to speak concurrently with more relatives.^29,33^

Nonetheless, maintenance of an individual and person-centred approach during care planning discussions appeared to be challenged by remote communication. The most commonly reported challenges included difficulty understanding and monitoring emotions, knowing how to introduce sensitive topics for the first time in the absence of face-to-face contact and non-verbal cues.^30,33,35^ The absence of non-verbal communicative mechanisms contained in face-to-face interactions, combined with relatives’ inability to monitor their loved one themselves tended to generate an erosion of relationships and trust between care home staff, relatives and surrogates.

Although it was evident that face-to- face bedside discussions between care home staff and residents had frequently taken place with staff wearing full personal protective equipment (PPE),^29,33^ there was a notable a lack of data reporting on facilitatory mechanisms used during these encounters.

#### Training and education

Reflecting the realisation that new knowledge and skills were required to enable staff to engage effectively in new modes of delivery, and knowledge of COVID-19 and its care, four of the included studies referenced education and training associated with advance care planning during the COVID-19 pandemic. Synthesis identified several facilitatory mechanisms generated by education and training delivered during the pandemic.^29,30,32,34^

Training focused on equipping staff with the knowledge and confidence to be able to engage in advance care planning during the pandemic, for example, how to use remote communication effectively, how to support the emotional needs of relatives and developing staff’s understanding of COVID-19 and its care.^30,32,34^ Training was delivered via a range of methods which included online asynchronous sessions, websites, videos, to face to face scenario-based learning and on the job training such as mentoring/role modelling.^29,30,32,34^ In the case of role modelling and mentoring, external staff (such as nurse practitioners) were used to deliver training on a continuing ongoing basis (rather than a single delivery) in an effort to mitigate the high rates of staff sickness and shortages experienced during the pandemic.^29,32^ Most education tended only to be delivered to staff directly involved in advance care planning, with only one study including relatives and significant others in the training.^34^ It was evident that training and education prompted more conversations about advance care planning between care home staff and relatives and helped to relieve care home staff’s fears and misconceptions about COVID-19.^29,30,32,34^

### Human agency

Human agency represents how stakeholders interpret and respond to the identified intervention and contextual mechanisms. Three related themes were distilled from the literature: mitigation of the negative effects of reduced face-to-face interaction; changes in perceptions and cultures concerning preparation for death; and continuities and changes in the topics discussed in advance care planning conversations.

#### Optimising remote communication

Despite the erosion of trust that accompanied the replacement of face-to-face interaction with remote communication, there appeared to be a consistent understanding and acceptance of the need to communicate remotely despite the evident challenges.^29–31^ Several responses were identified by which care home staff acted to mitigate these challenges. These included engaging in more frequent discussions (treating care planning as ongoing rather than one-off discussions), active listening and spending longer informing relatives on their loved one’s condition and being more alert to emotions in the absence of non-verbal communication.^29,30,33,38^ Another important response was the sharing or transfer of responsibility to colleagues, such as home managers or nurse practitioners when discussions were perceived as not being effective.^29,30^ Referring to colleagues in this way appeared to facilitate an understanding of the importance of collaborative teamwork and togetherness, for example more experienced staff supporting younger or less experienced staff through pandemic conditions.^29,31^

#### Changes in perceptions and cultures

It was evident that the pandemic led to cultural and perceptual changes towards advance care planning and talking about death and dying. Specifically, our synthesis suggested that the reality of death and decline brought by COVID-19 encouraged more care home staff, residents and relatives to want to prepare for the end of life, cultivating a more open culture and increased recognition of care planning.^30,31,33^ In terms of care home staff, this change was manifested in their triggering of earlier and more frequent discussions with residents and relatives,^33,38^ with changed communication patterns more conducive to regular contact between staff and relatives.^31^

Furthermore, a change in preference was noted in regard to relatives’ and residents’ opinions of hospitalisation and intensive care unit use at the end of life.^33,35,38^ Synthesis revealed an increased desire to avoid hospital admission with more residents and surrogates initiating ‘do not hospitalise’ orders compared to pre-pandemic.^35–38^ For residents the main driver behind this appeared to be a fear of contracting COVID-19, and for relatives a fear of their loved one dying alone.^29,30,33^ Despite this, when relatives were unaware of their loved one’s health condition and in cases of acute and unexpected decline (which can often be the case with COVID-19 deaths), they tended to doubt information given to them, express shock and were more reluctant to accept and discuss death.^30,35^

#### Topics of discussion

Stakeholders’ responses to the intervention mechanisms associated with the introduction of distance communication were largely consistent, in that the overarching advance care planning topics remained the same in remote conversations, with discussions centred around care preferences, prognosis, treatment goals and bereavement support. However, reflecting stakeholders’ responses to the contextual changes wrought by COVID-19, more anticipatory conversations were evident during the pandemic about the use of ventilation, intensive care unit admission as well as resuscitation and hospital usage at the end of life.^29,33,35^ For example, Ye et al.^38^ who studied care preferences amongst 963 residents and surrogate decision makers found that 276 changed their hospitalisation preferences to ‘do not hospitalise’ following COVID-19 discussion topics. Similarly, Vellani et al.^29^ report increased frequency of advance care planning discussions during the COVID-19 pandemic in care homes.

However, our synthesis suggests that holistic care planning discussions of multiple topics tended to be less evident in light of these more prominent topics of discussion related to COVID-19. Specifically, it was apparent that conversations could take a linear form related to singular topics such as expressing a wish to not be admitted to hospital due to a fear of catching COVID-19 and preferences around ventilation.^33,39^

### Outcomes

In consonance with the critical realist tenet that outcomes of interest should not be confined to rates of behaviour but should also encompass experiential consequences, in addition to reporting data on the frequency and completeness of advance care planning conversations, we discuss the effect of the mechanisms described above on stakeholders’ wellbeing. Specifically, we note that the literature indicates that mechanisms embedded in training and education interventions tended mitigate the negative effects on staff’s confidence that was caused by the challenges of COVID-19 and the consequent adoption of remote technologies. Notwithstanding these positive mechanisms, the adoption of modes of remote communication generated an additional emotional toll on those who engaged with them.

#### Frequency and completeness

Findings in relation to frequency and completeness were inconsistent across the studies reviewed. Several studies suggested that the need and frequency of advance care planning discussions increased during the pandemic,^29,33,37^ and more plans were being updated to accommodate changing preferences.^38^ However, increases in the frequency of discussions were not consistent across the data, with studies also reporting that fewer residents had been offered end of life care discussions during the pandemic.^35,36^ Moreover, Jones et al.^39^ found that planning documentation often lacked sufficient detail to fully inform care as despite residents from 136 homes who died from COVID-19 specifying their resuscitation wishes, only 46% had a detailed care plan in place. Reduced frequency and completeness of care plans was found to lead in some cases to uncertain and reactive decisions being made, even 7 months into the pandemic.^35,39^ Similarly, Gonella et al.^30^ identified that some staff in care homes had difficulty exploring relatives’ preferences for care at the end of life due to a lack of detail in the advance care directives.

#### Confidence and preparedness

It was consistently reported that education and training improved stakeholders’ confidence and preparedness for advance care planning during the pandemic.^29,32,34,37^ Results suggest that developing care home staff’s knowledge and understanding of advance care planning gave them the confidence to have ongoing discussions with relatives in pandemic conditions.^32,34^ It was also suggested that relatives’ confidence, acceptance and awareness of care planning was enhanced through developing their knowledge and understanding.^32,34^ However, sustainability of outcomes related to education and training in the longer term has yet to be determined.

#### Emotional toll

Conducting sensitive and personal discussions remotely was found to add to the existing emotional toll associated with advance care planning.^30,31,33^ Specifically, the reduction of face-to-face contact and non-verbal communication made it harder to share, express and recognise emotions and build trusting relationships.^30,33^ Despite the increased emotional toll, care home staff had reduced time due to workload to focus on self-care.^30^ Only one study in this review included information about self-care for care home staff.^34^

The reduced face-to-face contact during the pandemic also appeared to take an emotional toll on relatives and surrogates involved in care planning.^30,33,35^ Emotions such as denial and shock concerning a loved one’s condition (particularly in the case of acute COVID-19 diagnosis) seemed to be triggered by relatives’ reduced ability to directly observe changes over time.^30,31^ Although synthesis suggests these circumstances can lead to a greater risk of interventional and curative orientated decisions by relatives,^29,30,35^ it was found that frequent ongoing involvement of relatives in care planning conversations can help to mitigate these emotions.^29,30^

## Discussion

Using a critical realist lens to examine the literature on advance care planning in care homes highlights the temporal relationship between ever-changing social and physical contexts and people’s responses to them and facilitates the identification of mechanisms embedded in both these aspects of the social world. It thus allows for the development of a theoretical model that explains both the evolution of interventions and their effects within the social milieu to which they have been introduced.

The advent of COVID-19 created a context whereby three different types of mechanism interacted to potentiate a dramatic rise in death rates. These include: the strong vectors of infection that result from the physical organisation of care homes which involve large numbers of people living and working in proximity in partially closed institutions ^40^; residents’ vulnerability to the negative effects of infection^1,41^ generated by biological mechanisms associated with senescence and frailty; and a virus containing powerful mechanisms of transmissibility and virulence.^42^ The responsive actions taken by those responsible for the delivery of care in homes was to develop interventions designed to disrupt transmission of the virus, primarily the use of personal protective equipment in interactions between staff and residents and the closing of homes to visitors.^32,33^ The implementation of these types of interventions created, in turn, a new context which disrupted the face-to-face interactions between residents, close others and staff upon which effective advance care planning had previously depended. The responses of care designers to this novel context included the adoption of digitally based modes of remote communication, often accompanied by education and training in their effective use, which they hypothesised would mitigate the effects of this disruption.^30,34^

These interventions created yet another contextual iteration. The responses of those involved in advance care planning to this context were varied. The threat of the pandemic tended to encourage earlier and more frequent advance care planning discussions,^29,33^ though this tendency was partially countervailed by the difficulties that some residents had in engaging with new communication modes.^35^ Another complexity in response involved apparently contradictory trends in relation to curative versus palliative approaches. On one hand, there was an increase in the number of residents and relatives deciding against the option of hospitalisation, hospitals being associated with a higher probability of infection and a lonely death.^35–38^ Indeed, hospitalisation and associated topics of discussion, such as preferences concerning ventilation, often dominated conversations to the exclusion of other issues.^33,39^

On the other hand, a loss of trust that derived partially from the attenuation of communication links led to a tendency for relatives and surrogates to request curative interventions against professional advice when residents’ health conditions were unknown or declined rapidly and unexpectedly,^30^ as is often the case in COVID-19 infection.^42^ However, this tendency is not unique to COVID-19 or the care home population, Zhang^43^ found that patients with an acute serious illness and their relatives were often focused on curative treatments and survival, regardless of age or comorbidities. Similarly, Auriemma et al.^44^ found that relatives and patients who are still processing a new acute life-threating diagnosis may struggle to come to terms with their prognosis and may not be prepared to discuss end of life preferences.

The outcomes of these complex and temporally evolving intersections between generative mechanisms are equivalently complex, displaying often contradictory tendencies. So, on the one hand there was evidence that one of the outcomes of the introduction of remote communication in circumstances where death was perceived to be close was a tendency for relatives to have more frequent contact,^29,33^ while on the other hand, a tendency for relatives and residents to focus on singular topics (such as resuscitation and ventilation) at a cost to more holistic approaches required for deeper relationships tended to mean that planning was often incomplete.^30,39^ This, combined with relatives’ lack of confidence in staff’s decisions, compromised the utility of plans to inform care. However, there was evidence that these negative effects could be mitigated by education and training in advance care planning using remote technology, which increased staff’s confidence and readiness to engage with these new modes.^34^ It therefore encouraging that ‘COVID centric’ training and education interventions are being developed in this area,^45^ and it is hoped our results can be used to further inform these future projects.

While education and training may have eased the psychological burden on staff, it is important not to underestimate the weight of that burden. The communication difficulties associated with remote technologies meant that concerns about engaging effectively with relatives further exacerbated the emotional toll of dealing with high death rates in circumstances where staff shortages stretched the capacity of those remaining to provide essential care, including advance care planning to the limit.^30–32^ Similar communication difficulties have been reported in general practice and in the hospice sector, with findings suggesting digital communication created a separation and made sensitive conversations more difficult.^46,47^ In the iterative flow of social life, all of these outcomes now stand as part of the new context facing current staff, thus indicating the next round of intervention development required to maintain and improve the effectiveness of advance care planning in care homes.

### Strengths and limitations

It is recognised this review included data collected from a range of different care home types and sizes from different countries, which also had different and changing responses to the COVID-19 pandemic. However, the heterogeneity of the included data was a strength in that it was necessary to identify a range of underlying mechanisms to provide a foundation for a deeper understanding of what works, for whom and in what circumstances. As with all realist syntheses, judgements had to be made on the inferences within the included data, however we aimed to report all steps in our synthesis process to support transparency and reproducibility, as well as to inform the evaluation and development of realist synthesis. The authors acknowledge that this review is framed around a part of the pandemic, and that synthesis of future work can expand and develop on the presented results. Lastly, restricting the search to English language may have led to some relevant studies being excluded.

## Conclusion

This review has evidenced that communication difficulties associated with remote technologies, increased exposure to sensitive discussions about death and dying in a context of chronic workforce shortages placed unsustainable emotional pressures and expectations on the care home workforce. Furthermore, the novel symptoms and trajectories of decline associated with COVID-19 combined with reduced visits to observe residents challenged the capacity to plan for future care with some relatives having difficulty accepting their loved one’s decline. Despite these challenges, evidence suggests that education and training in advance care planning increased care home staff’s confidence and readiness to engage in care planning during pandemic conditions.

Opportunities were also generated by pandemic conditions. Specifically, the introduction of remote communication in circumstances where death was perceived to be close was a stimulus for relatives to have more frequent and earlier care planning conservations. Moreover, an increase in the number of residents and relatives deciding against the option of hospitalisation was evident.

In sum, these results highlight part of the new context facing staff, relatives and residents in care homes, thus providing valuable insight for future intervention development required to maintain and improve the effectiveness of advance care planning in care homes during and beyond the pandemic.

## Supplemental Material

sj-pdf-1-pmj-10.1177_02692163221137103 – Supplemental material for Understanding advance care planning in care homes throughout the COVID-19 pandemic: A critical realist review and synthesis Click here for additional data file. Supplemental material, sj-pdf-1-pmj-10.1177_02692163221137103 for Understanding advance care planning in care homes throughout the COVID-19 pandemic: A critical realist review and synthesis by Adam Spacey and Sam Porter in Palliative Medicine
