# Supplementary material for: Understanding advance care planning in care homes throughout the COVID-19 pandemic: A critical realist review and synthesis
Source: Palliat Med. 2022 Nov 12;37(5):663–76. doi: 10.1177/02692163221137103 (PMC9659704; doi:10.1177/02692163221137103)
Supplement: sj-pdf-1-pmj-10.1177_02692163221137103 – Supplemental material for Understanding advance care planning in care homes throughout the COVID-19 pandemic: A critical realist review and synthesis [file sj-pdf-1-pmj-10.1177_02692163221137103.pdf]

How to use this Excel spreadsheet on the Mixed Methods Appraisal Tool (MMAT)?

1 Enter information on the papers you are appraising.

- RefID: if you have several papers to appraise, adding a reference number can be useful.
- First author: family name of the first author.
- Year: year of publication of the paper.
- Citation: full reference of the paper including title, journal, volume and pages.

2 Respond to the two screening questions.

- Responding 'No' or 'Can't tell' to one or both questions might indicate that the paper is not an empirical study, and thus cannot be appraised using the MMAT.

3 Choose the appropriate categories of studies to appraise.

- For a qualitative study: choose the qualitative set of criteria (set #1).
- For a quantitative study: choose the appropriate quantitative set of criteria (either set #2, 3 or 4).
- For a mixed methods study: choose three sets of criteria, i.e., the qualitative set (set #1) the appropriate quantitative set (either set #2, 3 or 4) and the mixed methods set (set #5).

4 Rate the criteria of the chosen category (or categories).

- 'Yes' means that the criterion is met.
- 'No' means that the criterion is not met.
- 'Can't tell' means that there is not enough information in the paper for you to judge if the criterion is met or not.

For more information: http://mixedmethodsappraisaltoolpublic.pbworks.com

ID	First author	Year	SCREENING QUESTIONS S1. Are there clear research questions?	S2. Do the collected data allow to address the research questions?
	Berning et al.	2021	Yes	Yes
	Strang et al.	2021	Yes	Yes
	Vellani et al.	2021	Yes	Yes
	Gonella et al.	2022	Yes	Yes
	Hockley et al.	2021	Yes	Yes
	McGilton et al.	2021	Yes	Yes
	Brugge et al.	2022	Yes	Yes
	Ye et al.	2021	Yes	Yes
	Jones et al.	2022	Yes	Yes
	Hack et al.	2022	Yes	Yes
	Cousins et al.	2022	Yes	Yes

1. QUALITATIVE STUDIES			
1.1. Is the qualitative approach appropriate to answer the research question?	1.2. Are the qualitative data collection methods adequate to address the research question?	1.3. Are the findings adequately derived from the data?	1.4. Is the interpretation of results sufficiently substantiated by data?
Yes	Yes	Yes	Yes
Yes	Yes	Yes	Yes
Yes	Yes	Yes	Yes
Yes	Yes	Yes	Yes
Yes	Yes	Yes	Yes
Yes	Yes	Yes	Yes

	2. RANDOMIZED CONTROLLED TI		
1.5. Is there coherence between qualitative data sources, collection, analysis and interpretation?	2.1. Is randomization appropriately performed?	2.2. Are the groups comparable at baseline?	2.3. Are there complete outcome data?
Yes			
Yes			
Yes			
Yes			
Yes			
Yes			

RIALS			3.
2.4. Are outcome assessors blinded to the intervention provided?	2.5 Did the participants adhere to the assigned intervention?	3.1. Are the participants representative of the target population?	3.2. Are measurements appropriate regarding both the outcome and intervention (or exposure)?

NON-RANDOMIZED STUDIES			
3.3. Are there complete outcome data?	3.4. Are the confounders accounted for in the design and analysis?	3.5. During the study period, is the intervention administered (or exposure occurred) as intended?	4.1. Is the sampling strategy relevant to address the research question?
			Yes
			Yes
			Yes
			Yes

4. QUANTITATIVE DESCRIPTIVE STUDIES			
4.2. Is the sample representative of the target population?	4.3. Are the measurements appropriate?	4.4. Is the risk of nonresponse bias low?	4.5. Is the statistical analysis appropriate to answer the research question?
Yes	Yes	Yes	Yes
Yes	Yes	Yes	Yes
Yes	Yes	Yes	Yes
Yes	Yes	Yes	Yes

5. MIXED METHODS STUDIES			
5.1. Is there an adequate rationale for using a mixed methods design to address the research question?	5.2. Are the different components of the study effectively integrated to answer the research question?	5.3. Are the outputs of the integration of qualitative and quantitative components adequately interpreted?	5.4. Are divergences and inconsistencies between quantitative and qualitative results adequately addressed?
Yes	Yes	Yes	Yes

5.5. Do the different components of the study adhere to the quality criteria of each tradition of the methods involved?
Yes
